# Redox Disruption Induced by Saquayamycin B1 Promotes Cytotoxicity in Resistant Melanoma Cells

**DOI:** 10.1002/cmdc.70308

**Published:** 2026-05-22

**Authors:** Geovana Guedes Silvestre, Thalisson Amorim de Souza, Alan Ferreira Alves, Valeria Dutan‐Patiño, Jean‐Michel Huvelin, Mathilde Gourdel, Mikael Croyal, Samuel Cibulski, Demetrius Antonio Machado de Araújo, Marcus Tullius Scotti, Josean Fechine Tavares, Angela Tesse, El‐Hassan Nazih, Marianna Vieira Sobral

**Affiliations:** ^1^ Postgraduate Program of Bioactives Natural and Synthetics Products PgPNSB Federal University of Paraíba João Pessoa Brazil; ^2^ UR2160 ISOMER Nantes University Nantes France; ^3^ CHU Nantes SFR Santé Inserm UMS 016 CNRS UMS 3556 Nantes University Nantes France; ^4^ FACISA Federal University of Rio Grande do Norte Santa Cruz Brazil; ^5^ CBIOTEC Biotechnology Center Federal University of Paraíba João Pessoa Brazil; ^6^ UMR Inserm 1235 TENS Nantes University Nantes France

**Keywords:** angucyclines, electronic paramagnetic resonance (EPR), glutathione S‐transferase pi 1‐1 (GSTP1‐1), oxidative stress, S*treptomyces*

## Abstract

Melanoma is an aggressive skin cancer characterized by rapid progression and frequent chemoresistance, which limits the success of current therapies. Saquayamycin B1 (SQ‐B1), an angucycline isolated from *Streptomyces* sp. I072, was investigated for its antimelanoma activity with emphasis on redox disruption. The compound was purified using M9 fermentation followed by semipreparative liquid chromatography, and its structure was confirmed through 1D/2D nuclear magnetic resonance (NMR) and mass spectrometry. Cytotoxicity assays (MTT, 72 h) in SK‐MEL‐5, −113, −117, and −134 cells revealed IC_50_ values between 5.01 and 0.99 μM. Pretreatment with N‐acetylcysteine (NAC) rescued cell viability at IC_50_ and sub‐IC_50_ concentrations, supporting a ROS‐dependent mechanism. SQ‐B1 (4 μM, 24 h) reduced the GSH/GSSG ratio (3.49  ±  0.70 vs. 12.35  ±  0.27 in controls) and increased O^2•−^ and NO^•^ levels, indicating marked oxidative imbalance. Molecular docking suggested strong binding of SQ‐B1 to glutathione transferase (GSTP1‐1; *p* ≈ 0.86) and glutathione reductase (GSR; *p* ≈ 0.97), with weaker affinity for GPx4 (*p* ≈ 0.35). Molecular dynamics confirmed stable interactions between SQ‐B1 and GSR (RMSD plateau ~10 ns) with a total interaction energy of −201.0 kJ/mol. These findings indicate that SQ‐B1 induces cytotoxicity through targeting of key redox regulators, highlighting its potential as a redox‐modulating candidate for chemoresistant melanoma.

## Graphical abstract

Saquayamycin B1 (SQ‐B1), an antibiotic isolated from *Streptomyces* sp. I072, triggers cell death in different melanoma cell lines, including chemoresistant SK‐MEL‐5. SQ‐B1 elevates O_2_^•‐^ and NO^•^ on both sides of the membrane and blocks glutathione transferase (GSTP‐1) and glutathione reductase (GSR), destabilizing the GSH/GSSG antioxidant system. This imbalance underlies the marked cytotoxicity observed in chemoresistant melanoma cells.

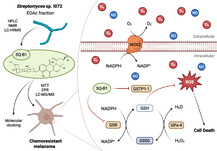

## 1 Introduction

Natural products of bacterial origin have long played a central role in cancer therapy. Among them, anthracyclines are well established as chemotherapeutic agents due to their potent cytotoxic effects, primarily mediated through the inhibition of topoisomerase II and intercalation into DNA, as well as induction of oxidative stress and DNA damage [1]. Closely related to this class, angucyclines represent the largest group of aromatic polyketides and display a wide spectrum of biological activities, including cytotoxic, antimicrobial, and antiviral properties, making them promising scaffolds for drug discovery [2, 3]. These compounds are predominantly biosynthesized by actinobacteria of the genus *Streptomyces*, which are renowned for being the most prolific natural source of clinically relevant antibiotics and antitumor agents [4].

Recent reviews highlight the continued discovery of angucyclines and angucyclinones—over 280 compounds described between 1965 and 2023—including those derived from marine and terrestrial actinomycetes with promising therapeutic properties [2]. Among them, saquayamycin B1 (SQ‐B1) exhibits pronounced cytotoxicity against multiple human cancer cell lines, including prostate [5], breast [6], and colorectal cancer [7]. SQ‐B1 was first described as a non‐natural derivative obtained by mild acid hydrolysis of saquayamycin B [8], but later it was isolated as a natural product from two different *Streptomyces* sp. strains [5, 6]. Notably, the colorectal cancer study represents the first and only study to date exploring the molecular mechanism of its cytotoxicity, presenting pro‐apoptotic and antimigration/invasion effects through inhibition of the PI3K/AKT signaling pathway [7]. These reports underscore the substantial therapeutic potential of SQ‐B1 as an anticancer agent.

Skin cancer is one of the five most common cancers worldwide [9] and remains a major health concern due to its rising incidence. Melanoma, a malignant tumor derived from melanocytes, is the most aggressive form because of its rapid progression and high metastatic potential [10]. In 2022, an estimated 331,722 new melanoma cases and 58,667 related deaths were reported globally, reflecting its high mortality rate [11]. The ability of melanoma to spread to vital organs such as the liver, lungs, brain, and bones leads to a markedly poor prognosis once distant metastasis occurs. While targeted therapies (e.g., BRAF, NRAS, KIT inhibitors) and immunotherapies, particularly checkpoint inhibitors, have improved survival, a significant proportion of patients exhibit intrinsic or acquired resistance, limiting long‐term efficacy [12].

Moreover, melanoma shows considerable chemoresistance, with conventional chemotherapy offering minimal clinical benefit. These therapeutic limitations reinforce the urgent need for novel treatment strategies capable of overcoming resistance, targeting less common molecular drivers, and achieving more durable responses. In this context, alternatives such as photodynamic therapy and agents that enhance oxidative stress in tumor cells are being actively investigated as promising approaches [13].

Melanoma cells display adaptive mechanisms that enable them to withstand the effects of elevated levels of reactive oxygen and nitrogen species (ROS and RNS, respectively) [14]. However, when ROS levels exceed the cellular antioxidant buffering capacity, oxidative damage accumulates and cell death pathways may be activated [15]. In this context, oxidative stress plays a dual and complex role in melanoma pathophysiology. On one hand, increased ROS levels contribute to DNA damage, raise mutation rates, and promote tumor progression, angiogenesis, and metastasis. On the other hand, this redox imbalance can be exploited therapeutically, as further ROS accumulation may induce selective cytotoxicity in tumor cells [16].

Here, we describe the SQ‐B1 isolation from *Streptomyces* sp. I072, a strain obtained from the Brazilian semiarid biome. Furthermore, we evaluated the cytotoxicity against four melanoma cell lines and started an in vitro investigation of the antimelanoma activity of SQ‐B1 in SK‐MEL‐5 cells, the chemoresistant line. Our results are consistent with a SQ‐B1 pro‐oxidant mechanism, which was evidenced by increased levels of both superoxide anion (O^2•‒^) and nitric oxide (NO^•^), as well as the modulation of the reduced‐to‐oxidized glutathione ratio (GSH/GSSG).

## 2 Results and Discussion

Preliminary exploratory analysis of the EtOAc‐F obtained from *Streptomyces* sp. I072 strain revealed the presence of a major compound in the sample. Based on that, the fraction was then subjected to semipreparative HPLC separation (Figures S1‐S3). The major peak was isolated and obtained in the form of a reddish solid, with a yield of 30 mg. Their molecular formula was determined by HRMS as C_31_H_33_O_12_, compatible with m/z 597.1954 [M + H] (calc. 597.1966, error 2.0 ppm). In addition, the chemical shifts of ^1^H and ^13^C spectra (Table 1) and, the correlation maps of 2D nuclear magnetic resonance (NMR) experiments were also analyzed (Figures S4‐S9). Altogether, the acquired data and comparison with literature [5] led to the identification of saquayamycin B1.

**TABLE 1:** ^1^H and ^13^C NMR (400 and 100 MHz, CDCl_3_) assignments of SQ‐B1, δ in ppm.

Position	Experimental		**Reference** ^**a**^	
	**δ**_**C**_	**δ**_**H**_	**δ**_**C**_	**δ**_**H**_
1	205.1		205.1, C	
2	52.3	2,60, m, 2,94, dd	52.6, CH_2_	2.60, d, 2.93, dd
3	76.3		76.3, C	
3‐CH_3_	30.4	1.27, s	30.5, CH_3_	1.27, s
3‐OH		3.90, brs		3.90, brs
4	43.4	1.83 d, 2.26 dd	43.4, CH_2_	1.82, d, 2.25, dd
4ª‐OH	80.7	3.61, brs	80.7, C	3.54, brs
5	144.5	6.39, d	144.5, CH	6.38, d
6	117.7	6.88, d	117.7, CH	6.87, d
6a	138.9		139.0, C	
7	188.2		188.2, C	
7a	114.1		114.2, C	
8‐OH	158.2	12.25, s	158.2, C	12.26, s
9	138.2		138.2, C	
10	134.0	7.87, d	134.0, CH	7.87, d
11	120.0	7.59, d	120.0, CH	7.59, d
11a	130.6		130.6, C	
12	182.2		182.2, C	
12a	138.3		138.3, C	
12b‐OH	76.2	4.95, brs	76.3, C	4.98, brs
**Sugar A,** **β‐D‐olivose**				
1′	71.6	4.92, brd	71.6, CH	4.94, brd
2′	36.9	1.43, m, 2,42ddd	36.9, CH_2_	1.40, m, 2.42, ddd
3′	76.9	3.78, m	76.9, CH	3.79, m
4′	74.6	3.48	74.7, CH	3.46, dd
5′	74.8	3.55, m	74.8, CH	3.55, m
6′	17.6	1.38, d	17.7, CH_3_	1.38, d
**Sugar B,** **α‐L cinerulose**				
1″	91.6	5.16, d	91.6, CH	5.16, d
2″	71.3	4.31, brq	71.3, CH	4.32, brq
3″	40.1	2.62, 2.64, dd,	40.2, CH_2_	2.60, dd, 2.66, dd
4″	207.9	—	208.0, C	—
5″	77.9	4.70, q	78.0, CH	4.70, q
6″	16.3	1.36, d	16.4, CH_3_	1.35, d

a Shaaban KA, Ahmed TA Leggas M, Rohr J., 2012.

Saquayamycin B1 induced cytotoxicity in different melanoma cell lines (Table 2). The least cytotoxic effect after 24 h was observed on the SK‐MEL‐113 cell line, IC_50_ of 11.45 ± 2.32 μM, while SK‐MEL‐117, 134 and 5 showed IC_50_ of 2.68 ± 0.29 μM, 2.06 ± 0.8, and 4.01 ± 0.55, respectively. Although SK‐MEL‐5 does not exhibit the highest sensitivity to SQ‐B1 in this treatment time, it is notable for its chemoresistance. This human melanoma cell line, derived from a lymph node of a young female patient, carries the BRAF V600E mutation, a key driver in melanoma metastatic progression. Additionally, SK‐MEL‐5 shows relatively high expression of the ABCB1 gene, which is involved in drug efflux mechanisms contributing to resistance against various chemotherapeutic agents. Therefore, SK‐MEL‐5 remains widely used in research to study tumor invasion, drug response, and molecular mechanisms underlying chemoresistance in melanoma [17].

**TABLE 2:** Cytotoxicity of SQ‐B1 against human melanoma cell lines and HaCat nontumor cell line after 24, 48, and 72 h of treatment, by MTT assay.

	24 h		48 h		72 h	
**Cell lines**	**IC**_**50**_ **,** **µM**	**SI**	**IC**_**50**_ **,** **µM**	**SI**	**IC**_**50**_ **,** **µM**	**SI**
SK‐MEL‐5	4.01 ± 0.55	1.23	2.24 ± 0.12	1.70	0.99 ± 0.14	3.93
SK‐MEL‐113	11.45 ± 2.32	2.30	6.91 ± 0.45	0.55	5.01 ± 0.45	0.77
SK‐MEL‐117	2.68 ± 0.29	1.85	2.53 ± 0.24	1.51	1.03 ± 0.09	3.78
SK‐MEL‐134	2.06 ± 0.07	2.41	1.67 ± 0.06	2.29	1.81 ± 0.40	2.15
HaCat	4.97 ± 0.41		3.83 ± 0.26		3.90 ± 0.27	

*Note:* Data obtained from three independent experiments (*n* = 3) were carried out in duplicate and presented as IC_50_ values obtained by nonlinear regression with a 95% confidence interval and expressed as mean ± standard error of the mean (SEM); SK‐MEL‐5, SK‐MEL‐113, SK‐MEL‐117, SK‐MEL‐134: human melanoma cell lines; HaCat: human immortalized keratinocytes cell line; CI_50_: mean inhibitory concentration; SI: selectivity index (IC_50_ nontumor cell line/IC_50_ tumor cell line).

Here, we investigated the cytotoxicity of SQ‐B1 associated with its pro‐oxidant effects, given that this mechanism plays a critical role in melanoma by disrupting the balance between ROS and cellular antioxidant defenses. Melanoma cells develop adaptive responses to survive elevated ROS levels, which at controlled concentrations promote genetic mutations and tumor progression, thereby supporting metastasis as well as treatment resistance [16]. Nevertheless, excessive accumulation of ROS can trigger cell death by damaging essential biomolecules and activating programed cell death pathways such as apoptosis and autophagy [18].

The mechanistic pathways underlying the cytotoxicity of SQ‐B1 remain unexplored. To date, the only report describing its mechanism of cell death indicates that SQ‐B1 induces apoptosis in colorectal cancer cells, mainly through modulation of the PI3K/Akt signaling pathway [7]. Evidence from other members of the angucycline class also supports the involvement of apoptotic mechanisms. For example, Landomycin E (LE) has been shown to induce programed cell death in Jurkat T‐leukemia cells through multiple signaling pathways, including the early, ROS‐independent activation of procaspase‐10 associated with receptor‐mediated apoptosis, as well as mitochondrial activation of apoptosis‐inducing factor (AIF) [19]. In addition, LE‐induced cell death has been associated with rapid hydrogen peroxide (H_2_O_2_) generation, suggesting a role for ROS in the death‐inducing process [20]. Based on these observations, it is widely accepted that further studies are warranted to characterize the potential pro‐apoptotic activity of SQ‐B1 and its possible relationship with oxidative stress‐mediated pathways.

**FIGURE 1:**
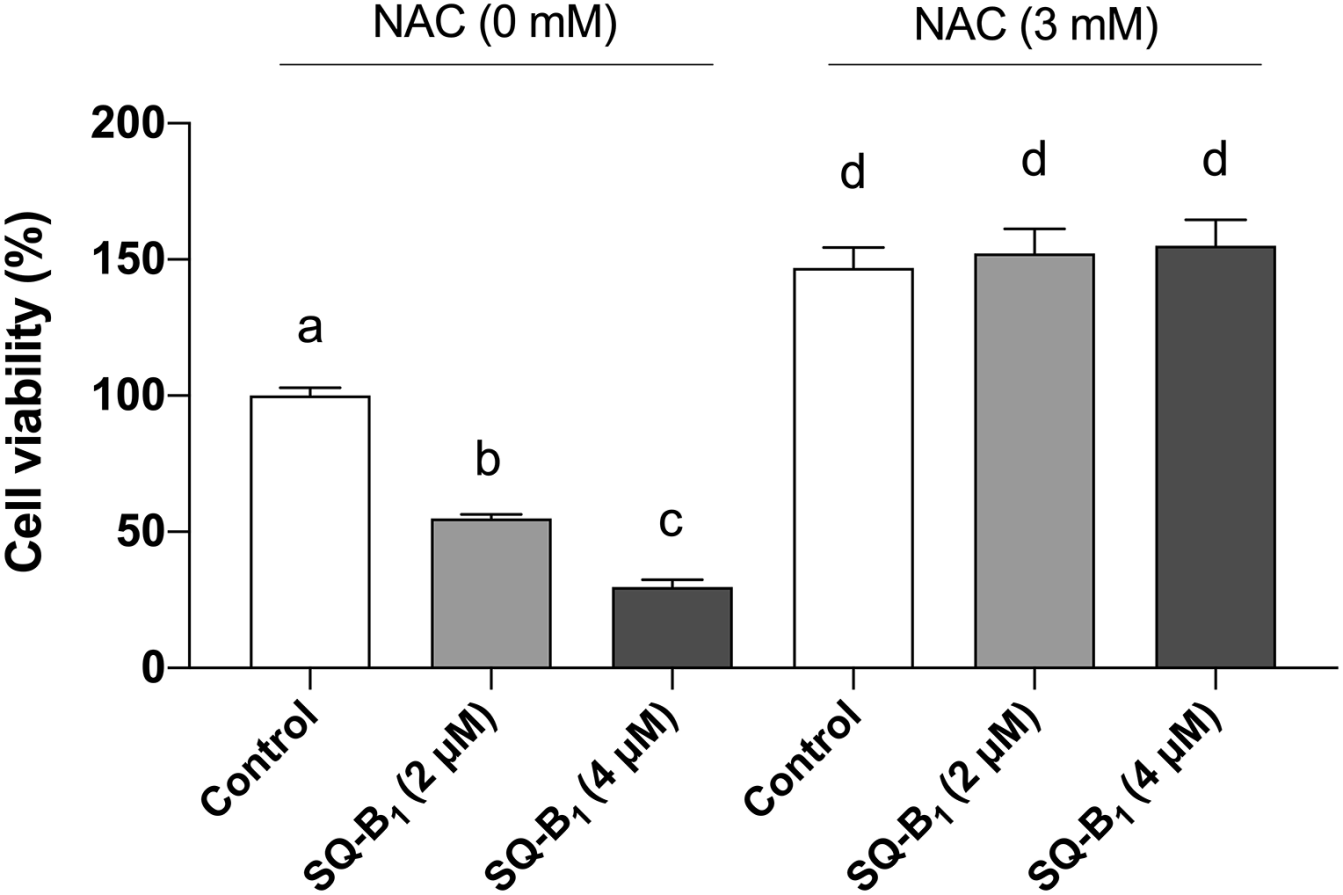
Cytotoxic effect of SQ‐B1 in SK‐MEL‐5 cells pretreated or not with NAC. MTT assay results expressed as percentage of cell viability (%), obtained from three independent experiments (*n* = 3) performed in triplicate. Data were analyzed by one‐way ANOVA followed by Tukey’s test. Different letters denote significant differences among conditions (*p* < 0.05).

To determine whether the cytotoxicity induced by SQ‐B1 is mediated by oxidative stress, an MTT assay was conducted in the presence of NAC, which works primarily as a cysteine prodrug, elevating intracellular cysteine concentrations essential for glutathione biosynthesis. Additionally, NAC exhibits antioxidant effects by neutralizing free radicals and facilitating the regeneration of thiol groups, thereby protecting cells from oxidative damage [21]. Pretreatment with NAC completely prevented the decrease in cell viability (Figure 1) observed in cells exposed to the IC_50_ concentration of SQ‐B1 (4 µM) (30.30% ± 4.36% viability without NAC vs. 171.9% ± 2.54% with NAC) and half the IC_50_ SQ‐B1 (2 µM) (54.25% ± 1.24% without NAC vs. 172.0% ± 1.13% with NAC), relative to the control group (100% viability), providing evidence that oxidative stress plays a central role in the cytotoxic mechanism of SQ‐B1.

**FIGURE 2:**
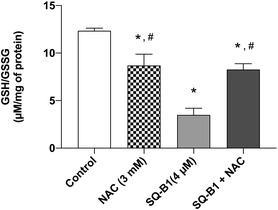
Effect of SQ‐B1 on GSH/GSSG (reduced to oxidized) levels in SK‐MEL‐5 cells measured after 24 h treatment. Quantification of the GSH/GSSG ratio by mass spectrometry. Values were normalized to protein content and expressed as µM/mg of protein. Data were obtained from two independent experiments (*n* = 2) performed in duplicate and were analyzed by one‐way ANOVA followed by Tukey’s test (*p < 0.05 vs. control; #*p* < 0.05 vs. SQ‐B1 4 µM).

There are several mechanisms regulating cellular oxidative stress that involve antioxidant agents capable of neutralizing reactive oxygen species and maintaining redox homeostasis, being the glutathione (GSH) system one of the most important components. GSH is a critical intracellular antioxidant tripeptide composed of glutamate, cysteine, and glycine, synthesized through a two‐step ATP‐dependent enzymatic process involving glutamate‐cysteine ligase and glutathione synthetase. During oxidative stress, two GSH molecules oxidize to form glutathione disulfide (GSSG), which can be enzymatically reduced back to GSH by glutathione reductase (GSR) using NADPH. The ratio of reduced GSH to oxidized GSSG serves as a sensitive biomarker for cellular redox status, with a high GSH/GSSG ratio indicating a reductive environment and a low ratio reflecting oxidative stress [22]. Thus, we measured GSH and GSSG levels in SK‐MEL‐5 cells after SQ‐B1 treatment for 24 h (Figure 2). Data showed a significant reduction in the GSH/GSSG ratio in cells treated with 4 µM (3.49 ± 0.70  µM/µg protein) compared to the control (12.35 ±  0.27 µM/µg protein). This result supports the pro‐oxidant effect of SQ‐B1.

**FIGURE 3:**
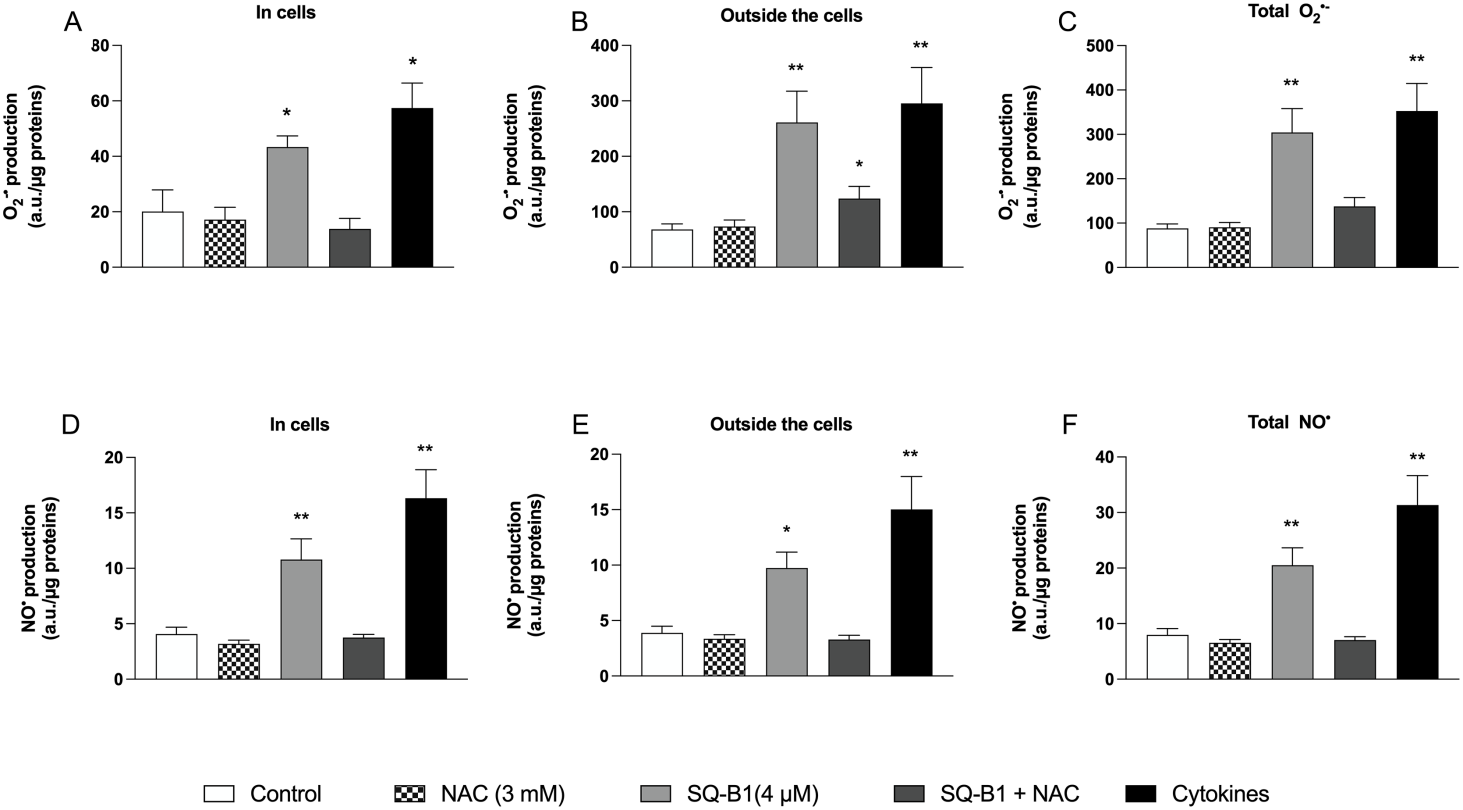
Effect of SQ‐B1 on superoxide anion (O_2_•^‒^) and nitric oxide (NO•) production in SK‐MEL‐5 cells following 24 h treatment with NAC (3 mM), SQ‐B1 (4 µM), SQ‐B1 + NAC, or Cytokines (IL‐1β 10 ng/mL, TNF‐α 100 ng/mL, and IFN‐γ 10 ng/mL), used as a positive control. (A‐C) O_2_•^‒^ production measured inside the cells (A), outside the cells (B), and total O_2_•^‒^ levels (C). (D‐F) NO• production measured inside the cells (D), outside the cells (E), and total NO• levels (F) under the same experimental conditions. Data are expressed as arbitrary units (a.u.) normalized to µg of protein. Results represent four independent experiments (*n* = 4) performed in duplicate. Statistical analysis was performed using one‐way ANOVA followed by Tukey’s test (*p < 0.05, **p < 0.01 vs. control).

To test our hypothesis, the production of superoxide anion (O_2_^•‒^) and nitric oxide (NO^•^) in SK‐MEL‐5 cells was quantified both intracellularly and extracellularly (Figure 3) using electronic paramagnetic resonance (EPR) spectroscopy, a gold standard method to detect free radicals in biological samples [23]. As expected, cells exposed to the cytokine cocktail exhibited significantly elevated O_2_^•‒^ levels inside and especially outside the cells compared with the control group. Similarly, SQ‐B1 treatment significantly increased O_2_^•‒^ production inside and especially outside cells, compared with the controls, suggesting a pro‐oxidant effect of SQ‐B1 in this cancer cell line, because it was prevented by the antioxidant compound NAC (Figure 3). NAC alone did not significantly modify O_2_^‒^ levels in cells compared to controls (Figure 3A–C). Concerning NO^•^ levels, SQ‐B1 treatment induced increased levels of this free radical inside and outside the cells compared to control cells, and NAC prevented this increase (Figure 3D–F).

The marked elevation of extracellular O_2_^•‒^ compared to intracellular levels may result from membrane‐bound NADPH oxidase, particularly NOX2, that is activated in pro‐oxidant and pro‐inflammatory conditions [24]. In our study, this effect may be further amplified by SQ‐B1‐induced cytotoxicity, since a possible plasma membrane disruption during in vitro cell death may lead to the leakage of intracellular ROS into the extracellular space [25]. In contrast, NO^•^ levels increased similarly inside and outside the cells, reflecting a possible inflammatory origin due to inducible nitric oxide synthase (iNOS) expression under oxidative stress conditions, contributing to nitrosative signaling due to peroxinitrite production and cellular damage [26]. This aspect needs to be investigated in further studies. Such mechanisms are consistent with the redox activity of angucyclines. For instance, landomycin A rapidly induces ROS formation, including hydrogen peroxide (H_2_O_2_) and hydroxyl radicals (^•^OH), leading to oxidative stress and apoptosis, effects attenuated by specific scavengers [27].

We evidenced that SQ‐B1 increased O_2_^•‒^ and NO^•^ production, this was associated with GSH/GSSG ratio reduction. However, this alteration does not necessarily indicate that the compound directly targets components of the glutathione system, since GSH oxidation occurs naturally during neutralization of reactive species. Thus, increased free radical generation leads to greater GSH consumption and, subsequently, higher GSSG levels [28]. Nevertheless, it is important to investigate whether SQ‐B1 can modulate the glutathione antioxidant system through the interaction with key enzymes, such as glutathione S‐transferase (GST), which catalyzes the conjugation of GSH to electrophilic substrates for detoxification; glutathione peroxidase (GSP), which reduces peroxides using GSH as an electron donor, forming GSSG. Furthermore, GSR regenerates GSH from GSSG using NADPH. To investigate this hypothesis, we conducted molecular docking analyses between SQ‐B1 and these enzymes, to explore potential interactions capable to impact cellular redox balance [29].

**FIGURE 4:**
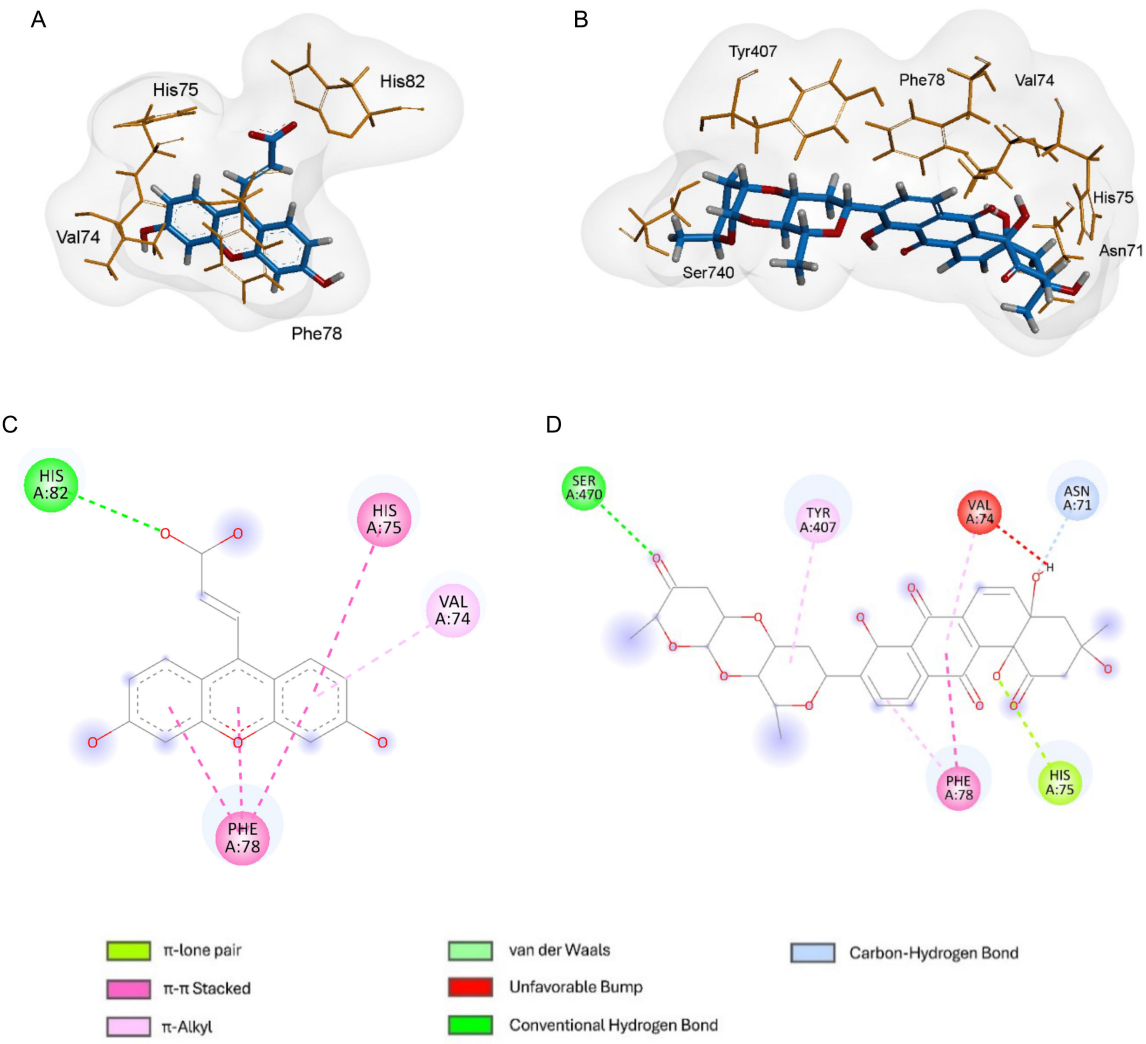
Molecular docking analysis of the compound with target proteins: 3D binding poses and 2D interaction maps. (A,B). 3D structures between 6‐hydroxy‐3‐oxo‐3*H*‐xanthene‐9‐propionic acid (A) and SQ‐B1 (B) against glutathione reductase. (C,D). Amino acid interactions between the same molecules.

Molecular docking is a computational approach to predict the most favorable binding conformation of a ligand within the active site of a target macromolecule or receptor. This technique can estimate the preferred binding pose and the strength of the interaction in a complex formed between a ligand and the target, where more negative values indicate more stable complexes [30]. The molecular docking results are presented in Table 3 evidencing the predicted probability (*p*) of the interaction, obtained by dividing the score of each compound against the lower score. The amino acid interactions and the 3D interactions of the GSR are represented in Figure 4, while the interactions with GPx4 and glutathione S‐transferase pi 1‐1 (GSTP1−1) are in Supplementary material (Figures S11, S12).

**TABLE 3:** Molecular docking scores and normalized probabilities (*p*) comparing SQ‐B1 and reference ligands against glutathione transferase, glutathione reductase, and glutathione peroxidase enzymes.

Protein	Ligand	Moldock score	(*p*)* **Moldock score**	Rerank score	(*p*)* **Rerank score**	PLANTS score	(*p*)* **PLANTS score**	(*p*)* **Total**
GSTP1‐1	PDBL^b^	−70.258	1.00	−58.857	0.99	−546.360	1.00	1.00
	SQ‐B1	−50.165	0.71	−59.422	1.00	−477.087	0.87	0.86
GSR	PDBL^a^	−58.357	0.86	−46.627	0.98	−547.320	1.00	0.95
	SQ‐B1	−68.037	1.00	−47.746	1.00	−500.822	0.92	0.97
GPx4	PDBL^c^	−232.706	1.00	−132.384	1.00	−529.025	1.00	1.00
	SQ‐B1	−44.1973	0.19	−49.327	0.37	−201.488	0.38	0.31

a 6‐(7‐nitro‐2,1,3‐benzoxadiazol‐4‐ylthio)hexanol.

b 6‐hydroxy‐3‐oxo‐3H‐xanthene‐9‐propionic acid.

c GX‐pep3.

* Normalized consensus value.

Regarding GSTP1−1, the docking simulations demonstrated that the complex formed with SQ‐B1 exhibited higher docking scores than the control ligand in two of the three scoring functions evaluated (Table 3). The control compound, 6‐(7‐nitro‐2,1,3‐benzoxadiazol‐4‐ylthio)hexanol, is a highly potent GSTP1−1 blocker with an IC_50_ of 0.8 μM. Despite the less favorable docking scores, SQ‐B1 still achieved a binding probability of 0.86, indicating a promising interaction profile.

An analysis of the amino acids residues revealed that SQ‐B1 and the reference ligand share three common residue interactions: Phe8, Val35, and Tyr108. While Val35 formed π‐alkyl interactions in both complexes, Tyr108 established a carbon‐hydrogen bond with SQ‐B1 that was not observed in the reference complex. Federici et al. (2009) reported the role of Ile104 as a steric barrier that restricts access of bulky ligands to the catalytic pocket [31]. Both SQ‐B1 and the reference inhibitor engage with Ile104, suggesting that SQ‐B1 may reproduce the inhibitory behavior of the reference ligand (Figure S11).

GSTs play a central role in detoxification and drug resistance, and their overexpression in melanoma has been linked to reduced drug accumulation, enhanced detoxification of electrophilic agents, as well as impaired apoptosis, all contributing to multidrug resistance [32]. Beyond their metabolic functions, GSTs also modulate signaling pathways involved in cell proliferation and death. GSTP1 is particularly relevant due to its regulatory function in intracellular signaling [33]. The GSTP1−1 isoform interacts with components of the MAPK signaling cascade, especially TRAF2, thereby modulating the transduction of signals mediated by TNF‐α and controlling JNK activation. These pathways are essential for cellular responses to external stimuli, oxidative stress, and apoptosis [34]. In this context, our docking results suggesting SQ‐B1 as a potential GSTP1 inhibitor highlight its possible use as an adjuvant strategy to sensitize melanoma cells to conventional therapies, especially considering the high levels of chemoresistance and the limited durability of responses to current targeted and immune‐based treatments [35].

Concerning GSR, the noncompetitive allosteric inhibitor 6‐hydroxy‐3‐oxo‐3*H*‐xanthene‐9‐propionic acid was used as the reference ligand. The simulations revealed lower docking scores for SQ‐B1 compared to the reference ligand. The predicted probability for SQ‐B1 was 0.97, slightly higher than that of the reference ligand, indicating a theoretically favorable binding profile. SQ‐B1 exhibited a similar profile to the other ligand regarding amino acids interactions: both formed interactions with His75, Phe78, and Val74, with the latter being an unfavorable one in the SQ‐B1‐GR complex. However, SQ‐B1 also exhibited a hydrogen bond with Ser470, which may counterbalance the unfavorable interaction and explain the lowest score seen compared to the reference inhibitor.

Since GR catalyzes the regeneration of GSH from its oxidized form (GSSG), its inhibition disrupts cellular redox homeostasis and promotes the accumulation of reactive species, a particularly harmful effect in tumor cells that heavily depend on antioxidant defenses [36]. In line with these predictions, molecular dynamics simulations confirmed stable binding of SQ‐B1 to GSR, and in vitro assays demonstrated a marked decrease in the GSH/GSSG ratio upon treatment, supporting its inhibitory activity. Thus, the dual targeting of GST and GSR by SQ‐B1 may sensitize cancer cells to oxidative stress and help overcome chemoresistance.

Finally, GPx4 was analyzed in complex with SQ‐B1 and GX‐pep3, a potent inhibitor with an IC_50_ of 10 μM [37]. The complex formed with the reference ligand exhibited lower docking scores across all scoring functions, with an affinity probability value of 0.31 compared to 1.0 for the reference peptide inhibitor. GX‐pep3 established a greater number of hydrogen bonds, which may account for the more favorable docking scores observed.

**FIGURE 5:**
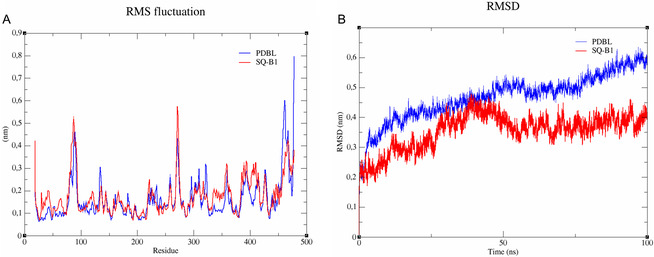
(A) Root mean square deviation (RMSD) of the protein backbone in complex with PDBL (blue) and SQ‐B1 (red) during 100 ns of molecular dynamics simulation. (B) Root mean square fluctuation (RMSF) of residues in the protein backbone for complexes with PDBL (blue) and SQ‐B1 (red) across the 100 ns trajectory.

To evaluate the stability of the protein‐ligand complexes, molecular dynamics simulations were performed for GR, which showed the best docking results among the tested targets, with SQ‐B1 and PDBL. The first parameter analyzed, RMSD (Figure 5A), measures the displacement of the protein and ligand over time. The simulations revealed that both complexes reached stability after approximately 10 ns of equilibration, with no evidence of structural unfolding throughout the trajectory. However, the PDBL complex showed greater deviations, while the SQ‐B1 complex maintained lower deviations (Figure 5B), indicating that SQ‐B1 provides greater structural stability to the protein by preserving a conformation closer to the initial docked structure compared with PDBL.

The interaction energy analysis of both complexes highlighted the differences in the binding nature of the two ligands. PDBL displayed a stronger total interaction energy (−443.637 kJ/mol), determined specially by electrostatic contributions (−359.250 kJ/mol) compared to van der Waals forces (−84.387 kJ/mol). However, despite the stronger total energy, these interactions were accompanied by larger energy fluctuations, indicating less stable interactions over the simulation. On the other hand, SQ‐B1 showed a lower total interaction energy (−201.04 kJ/mol), but with reduced fluctuations and energy drift, reflecting a more stable complex. The major contributor to the interaction energy was the van der Waals forces (−166.83 kJ/mol), while electrostatic interactions had a lower contribution (−34.22 kJ/mol). These results suggest that SQ‐B1 forms a more dynamically stable complex with the protein, whereas PDBL binds more strongly but induces greater structural perturbations, which may compromise the long‐term stability of the complex.

The RMSF profiles of both complexes showed similar fluctuation patterns, with most residues fluctuating within the range of 0.1–0.3 nm, consistent with a stably folded protein. SQ‐B1 induced slightly higher fluctuations at certain regions but maintained an overall stable backbone conformation. Together, these results indicate that both ligands form stable complexes with the protein, but SQ‐B1 exhibits enhanced stabilization of the global protein structure when compared to PDBL.

The molecular docking results indicate that the in vitro inhibitory activity of SQ‐B1 is most likely associated with interactions with GR and GSTP1−1, while GPx4 appears to be a less probable target. Consistent with these findings, the molecular dynamics simulations confirmed that SQ‐B1 forms a stable complex with GR. The lower root mean square deviation (RMSD) and RMSF values observed for SQ‐B1, compared with PDBL, further support its ability to stabilize the protein structure. Moreover, interaction energy analysis revealed that SQ‐B1 interactions are predominantly determined by van der Waals forces, and this complex has lower energy fluctuations compared to PDBL, indicating a more stable complex.

## 3 Conclusion

In summary, SQ‐B1, an angucycline isolated from *Streptomyces* sp. I072, exhibits potent cytotoxicity against human melanoma chemoresistant cell line, through a mechanism consistent with free radical production and oxidative stress induction associated with the modulation of redox‐regulating enzymes such as GSTP1−1 and GSR. The observed alterations in the GSH/GSSG balance and elevated O_2_^•−^ and NO^•^ levels support a pro‐oxidant and nitrosative effect that may sensitize melanoma cells to cytotoxic stress. Although further studies are required to elucidate the specific modes of cell death induced by SQ‐B1 and if this molecule is able to induce similar effects in other tumor cell lines. The findings of the present work demonstrate the potential of SQ‐B1 as a lead compound for melanoma therapy, suggesting its possible implication in the development of novel strategies against chemoresistant tumors.

## 4 Experimental Section

### 4.1 Fermentation and Metabolite Extraction of Streptomyces Sp. I072

*Streptomyces* sp. I072 was previously isolated from a soil sample collected in the Brazilian semiarid biome and identified as a member of the genus *Streptomyces* by MALDI‐TOF mass spectrometry (MS) analysis [38]. The strain was cultivated on soil‐agar medium at 37°C for 7–10 days until sporulation. Spore suspensions (1 × 10^7^/mL) were inoculated into 500 mL Erlenmeyer flasks containing M9 mineral medium (25% working volume) and incubated at 37°C for 14 days on a rotary shaker at 200 rpm. The resulting 1.5 L fermentation broth was filtered to separate the mycelium from the supernatant, which was extracted three times with an equal volume of ethyl acetate (EtOAc). The combined organic extracts were concentrated under reduced pressure using a rotary evaporator.

### 4.2 Isolation of Saquayamycin B1

Initially, high‐performance liquid chromatography (HPLC) analysis was carried out to evaluate the sample profile and complexity. The metabolites recovered in the EOAc fraction (EtOAc‐F) was dissolved in acetonitrile (ACN), and then subjected to exploratory analysis in analytical scale (Figures S1, S2). A gradient method was applied for the exploratory analysis, starting at 50% ACN and increasing to 100% over 60 min.

After method optimization (data not shown), the separations were scaled‐up and carried out by semipreparative HPLC with a LC‐20AR system (Shimadzu), SPD‐M40 diode array detector equipped with a C18 column (YMC‐Actus Triart, 250 mm  ×  20.0 mm i.d. filled with 5 µm particles), flow rate of 8 mL/min, and injection volume of 100 μL. A gradient elution starting from 60% (ACN) to 85% in 60 min was applied. The mobile phases were the same used for analytical analysis. Around 220 mg of EtOAc‐F was used in this step. After isolation, the major peak was dried under reduced pressure (BUCHI R‐300 equipped with vacuum pump model V‐100) and then subjected to 1D and 2D NMR experiments and liquid chromatography‐high‐resolution MS (LC‐HRMS) analysis, as herein described.

### 4.3 LC‐HRMS Analysis

A HPLC system coupled to a micrOTOF II MS (Bruker Daltonics, Billerica, MA, USA) coupled to an electrospray ion (ESI) source, was used for high‐resolution analyses as previously described [39]. In summary, the analysis parameters were as follows: capillary 4.5 kV, ESI in negative mode, final plate offset 500 V, 40 psi nebulizer, dry gas (N2) with a flow rate of 8 mL/min and a temperature of 200°C. The mass spectra (m/z 50‐1000) were recorded every 2 s. (Figure S3)

### 4.4 NMR Analyses

Uni and bidimensional NMR spectra (^1^H, ^13^C, DEPT, COSY, HMBC, and HSQC) were recorded on Bruker Avance III HD 400 MHz. The ^1^H and ^13^C NMR chemical shifts were referenced to the solvent peaks for CDCl_3_ (Figures S4‐S9) and compared with literature data [5].

### 4.5 Cell Lines and Culture

The melanoma cell lines SK‐MEL‐5, SK‐MEL‐113, SK‐MEL‐117 and SK‐MEL‐134 were purchased from the European Collection of Animal Cell Cultures (ECACC) (Salisbury, UK) and cultivated in Dulbecco’s Modified Eagle’s Medium (DMEM) or Roswell Park Memorial Institute Medium (RPMI) supplemented with 10% bovine fetal serum (BFS), 1% of L‐glutamine and 1% penicillin‐streptomycin. Cells were grown in a 5% CO_2_ humidified incubator at 37°C and culture maintenance was carried out every 48 h, using phosphate‐buffered solution (PBS), supplemented culture freshly prepared medium and trypsin. All the reagents cited were purchased from Sigma Aldrich (Saint‐Quentin Fallavier, France).

### 4.6 Cytotoxicity of Melanoma Cell Lines by MTT Assay

The cell viability assay was evaluated using the 3‐(4,5‐dimethylthiazol‐2‐yl)−2,5‐diphenyltetrazolium bromide (MTT) Sigma Aldrich (Saint‐Quentin Fallavier, France) test. Briefly, the cells were seeded in 96‐well plates (10,000 cells/well) and exposed to SQ‐B1 (1.56‐25 μM) for 24, 48 and 72 h in an atmosphere of 5% CO_2_ and 37°C. Controls were treated with a vehicle (DMEM + 0.5% DMSO). After incubation, the supernatant was removed, followed by the addition of MTT (10 mg/mL in PBS). After 4 h, formazan crystals were dissolved in DMSO and the absorbance was measured spectrophotometrically (SpectraMax 190, Molecular Devices, USA) at 570 nm. The half‐maximal inhibitory concentration (IC_50_) values were estimated after three independent experiments (*n* = 3) performed in triplicate. IC_50_ values were calculated by nonlinear regression using a four‐parameter logistic equation ($Y=\text{Bottom}+\frac{\text{Top}-\text{Bottom}}{1+10^{\left(\text{LogIC}50-X\right)\times \text{HillSlope}}}$) in GraphPad Prism (GraphPad Software, USA). The fitted curves are presented on the Supplementary material (Figure S10).

To evaluate the involvement of ROS in SQ‐B1 cytotoxicity, SK‐MEL‐5 cells were in 96‐well plates (10,000 cells/well) and exposed or not to 3 mM N‐acetylcysteine (NAC) Sigma Aldrich for 3 h. Subsequently, cells were treated with SQ‐B1 (2.0 or 4.0 μM) and incubated for 72 h as previously described. The MTT assay was performed as described in the previous paragraph.

### 4.7 Measurements of Free Radicals by Electronic Paramagnetic Resonance (EPR)

ROS and RNS production by SK‐MEL‐5 cells was quantified by EPR using a spin probe or spin trap respectively [40]. At this purpose, the cells were seeded in 24‐well plates (200,000 cells/well) and exposed to the follow conditions: (i) pool of pro‐inflammatory cytokines (IL‐β 10 ng/mL, TNF‐α 100 ng/mL and IFN‐γ 10 ng/mL) for 48h; (ii) SQ‐B1 (4.0 μM) for 24 h; (iii) NAC (3 mM); (iv) NAC (3 mM) and SQ‐B1 (4.0 μM); and (v) DMSO 0.2% as control. The plates were incubated in an atmosphere of 5% CO_2_ and 37°C. After treatment, the cells were washed and incubated with the spin‐probe or spin‐trap immediately prepared for 45 min.

For NO^•^ measurements a Krebs‐HEPES colloid solution containing Fe‐(DETC)_2_ was used as spin trap, prepared from 15 mM Na‐DETC (Sigma‐Aldrich, France) mixed to 8 mM FeSO_4_·7H_2_O (Sigma‐Aldrich, France). Then, for each sample we collected separately both spin trap and cells that were snap‐frozen in liquid nitrogen and analyzed in a Dewar flask at 77°K using a EPR Miniscope MS5000 (Bruker, Germany). The instrument settings to obtain NO^•^ spectra were: 10 mW microwave power; 1.0 mT amplitude modulation; 100 kHz modulation frequency; 120 s sweep time and three scans.

To measure O_2_^•^ production, SK‐MEL‐5 cells were incubated in a Krebs‐Hepes solution containing 1‐hydroxy‐3‐methoxycarbonyl‐2,2,5,5‐tetramethylpyrrolidine (CMH, 500 mM, Noxygen; Denzlingen, Germany) as spin probe, deferoxamine (25 mM, Sigma‐Aldrich, USA? Fr?), and diethyldithiocarbamate (DETC, 5 mM, Sigma‐Aldrich). Then, for each sample we collected separately both spin probe and cells that were snap‐frozen in liquid nitrogen before being analyzed. The parameters to obtain the spectra of oxidized CMH were as follows: microwave power: 10 mW; amplitude modulation: 0.600 mT; modulation frequency: 100 kHz; sweep time: 60 s; 3 scans.

Signals of both free radicals were quantified by measuring the amplitude of the peaks of the spectra obtained, expressed in arbitrary units (A.U.) and normalized to µg of total sample proteins measured using Pierce BCA Protein Assay Kits (ThermoScientific, Waltham, MA, USA).

### 4.8 GSH/GSSG Ratio Measurements

Oxidized (GSSG) and reduced (GSH) glutathione concentrations were determined by liquid chromatography‐tandem MS (LC‐MS/MS). All solvents used were LC‐MS grade and purchased from Biosolve (Valkenswaard, Netherlands). Standard compounds were obtained from Sigma‐Aldrich. A pool of reference standard solutions was prepared and serially diluted in an aqueous bovine serum albumin buffer (BSA, 3 g/L) to obtain seven standard solutions ranging from 0.1 to 100 μM. An exogenous internal standard ([^13^C_2_,^15^N]‐GSH; 50 μL, diluted to 50 μM in ultrapure water) was added to 50 μL of standard solutions and cell lysate samples. All samples were then treated with 300 μL of ACN to precipitate proteins. Samples were centrifuged for 10 min at 10,000 × g (10°C), and the supernatants were transferred to vials, dried under a gentle stream of N_2_, and resuspended in 5% ACN containing 0.1% formic acid for LC‐MS/MS analysis.

LC‐MS/MS analyses were performed on a Xevo TQD MS with an ESI interface coupled to an Acquity H‐Class UPLC system (Waters Corporation, Milford, MA, USA). Samples (10 μL) were injected onto an HSS T3 column (1.8 μm, 2.1 × 100 mm, Waters Corporation) held at 60°C. Compounds were separated using a linear gradient of mobile phase B (100% ACN, 0.1% formic acid) in mobile phase A (10% ACN, 0.1% formic acid) at a flow rate of 250 μL/min. Mobile phase B was maintained at 1% for 0.5 min, linearly increased from 1% to 3% over 2 min, then from 3% to 80% over 2 min, kept constant for 0.5 min, returned to the initial conditions over 0.5 min, and held for 1 min before the next injection. Targeted compounds were detected by the MS operating in positive ion mode (capillary voltage, 3 kV; desolvation gas [N_2_] flow and temperature, 1000 L/h and 400°C, respectively; source temperature, 120°C). Multiple reaction monitoring mode was applied for MS/MS detection: GSH, m/z 308.2 → 179.1 (cone/collision voltage, 25 V/12 eV); GSSG, m/z 613.3 → 355.1 (cone/collision voltage, 35 V/22 eV); [^13^C_2_,^15^N]‐GSH, m/z 311.2 → 182.1 (cone/collision voltage, 25 V/12 eV). Chromatographic peak area ratios between the unlabeled compounds and the internal standard were used to calculate detector responses.

Standard solutions were used to generate calibration curves for quantification. Data acquisition and integration were performed using MassLynx and TargetLynx software (Waters Corporation). Total protein content was quantified using a BSA standard curve. The results were normalized to protein content and expressed as µM per µg of protein (µM/µg protein).

### 4.9 Molecular Docking

For the in silico evaluation, Molegro Virtual Docker software v.2013.6.0.1 [41] was employed to perform the molecular docking and MarvinSketch software v.23.14 [42] was employed to model the tested molecule. The molecules were then optimized using the semiempirical MMFF method of Spartan v.14 [43].

The crystallographic structures of four targets were selected from the Protein Data Bank (PDB) to analyze if SQ‐B1 could potentially act through these targets. GSR (PDB ID: 1XAN) in complex with the noncompetitive inhibitor 6‐hydroxy‐3‐oxo‐3H‐xanthene‐9‐propionic acid [44]; Glutathione peroxidase 4 (GPx4) (PDB ID: 5H5S) in complex with peptide inhibitor GXpep‐3 [37] and Glutathione S‐transferase pi 1‐1 (GSTP1−1) (PDB ID: 3GUS) in complex with inhibitor 6‐(7‐nitro‐2,1,3‐benzoxadiazol‐4‐ylthio)hexanol [31].

Prior to the simulation, all water molecules and cofactors were excluded and a redocking step was performed to evaluate the accuracy and reliability of the results from the RMSD. The RMSD is a necessary step to verify that the algorithm was able to produce the correct pose, and values ≤2 Å are considered satisfactory. The analysis was conducted using the default settings of the software. The MolDock Score function was used to evaluate the ligand poses, considering internal energy, hydrogen bonds, and torsional energy contributions. Twenty runs were performed using the MolDock SE algorithm, and the top five poses were retained. A grid with a radius of 15 Å and a resolution of 0.30 Å was generated, centered on the positions of the crystallographic ligands on the selected proteins. The fitted poses were further analyzed using Discovery Studio Visualizer v21.1.0.20298 [45].

For each scoring function, the normalized score or predicted probability (*p*) was determined by dividing the score of each compound against the lowest score, as described as the following equation: *p* = (E_Lig_)/(E^min^_Lig_), where E_Lig_ represents the score of a ligand, and E^min^_Lig_ corresponds to the lowest energy observed among all ligands analyzed. The second consensus analysis consisted of calculating the overall average across all the scoring functions and calculate the mean of its probabilities, following the following equation: p(total) = (pMolDock score + pRerank score + pPLANTS score)/3. In this way, higher normalized scores indicate a greater predicted stability of the ligand‐target complex, with maximum value of 1.

### 4.10 Statistical Analyses

Statistical analyses were performed using GraphPad Prism 8.0.2 (Hearne Scientific Software, USA). Data normality was assessed using the Shapiro‐Wilk test, and homogeneity of variances was verified using Fisher’s test before performing one‐way ANOVA followed by Tukey’s multiple comparisons post hoc test. Data are expressed as mean ± standard error of the mean (SEM), and statistical significance was set at *p* < 0.05 or *p* < 0.01.

## Author Contributions

**Geovana Guedes Silvestre1**: conceptualization, data curation, writing (original draft). **Valeria Dutan‐Patiño**: cell culture, cytotoxicity analyses; **Jean‐Michel Huvelin**: protein measurements. **Samuel Cibulski** and **Thalisson Amorim de Souza**: SQ‐B1 obtention and chemical characterization. **Demetrius Antonio Machado de Araújo**: infrastructure and materials **Josean Fechine Tavares**: elucidation of SQ‐B1 chemical structure. **Alan Ferreira Alves** and **Marcus Tullius Scotti**: in silico analyses. **M. Gourdel** and **M. Croyal**: GSH/GSSG measurements. **Angela Tesse**: free radical measurements, writing (review and editing). **El‐Hassan Nazih**: supervision, writing (review and editing). **Marianna Vieira Sobral**: conceptualization, supervision, data curation, writing (review and editing).

## Funding

This study was supported by the Foundation of Research Support of Paraíba ‐ FAPESQ‐PB [11/2024].

## Conflicts of Interest

The authors declare no conflicts of interest.

## Supporting information

Supplementary Material

## Data Availability

The data that support the findings of this study are available from the corresponding author upon reasonable request.
