# Supplementary material for: Redox Disruption Induced by Saquayamycin B1 Promotes Cytotoxicity in Resistant Melanoma Cells
Source: ChemMedChem. 2026 May 22;21(10):e70308. doi: 10.1002/cmdc.70308 (PMC13206169; doi:10.1002/cmdc.70308)

Supplementary material

Redox Disruption Induced by Saquayamycin B1 Promotes Cytotoxicity in Resistant Melanoma Cells

Geovana Guedes Silvestre [a], Thalisson Amorim de Souza [a], Alan Ferreira Alves [a], Valeria Dutan-Patiño [b], Jean-Michel Huvelin [b], Mathilde Gourdel [c], Mikael Croyal [c], Samuel Cibulski [d], Demetrius Antonio Machado de Araújo [e], Marcus Tullius Scotti [a], Josean Fechine Tavares [a], Angela Tesse [f], El-Hassan Nazih [b], Marianna Vieira Sobral *[a]

[a] PgPNSB, Federal University of Paraíba, 58051-900, João Pessoa, Brazil.

[b] UR2160 ISOMER, Nantes University, 44322, Nantes, France.

[c] CHU Nantes, SFR Santé, Inserm UMS 016, CNRS UMS 3556, Nantes University, Nantes, France.

[d] FACISA, Federal University of Rio Grande do Norte, Santa Cruz, Brazil.

[e] CBIOTEC, Biotechnology Center, Federal University of Paraíba, João Pessoa, Brazil.

[f] UMR Inserm 1235 TENS, Nantes University, Nantes, France.

* Corresponding author. Department of Pharmaceutical Sciences of the Health Sciences Center, Federal University of Paraíba (UFPB), 58051-900, João Pessoa, Brazil. Phone: +55 (83) 3216-7200. E-mail: mariannavbs@gmail.com

Supplementary material

Figure S1 – Chromatographic profile of the EtOAc fraction of *Streptomyces* sp. I072 isolated from the soil of Cariri region, Paraíba - Brazil.

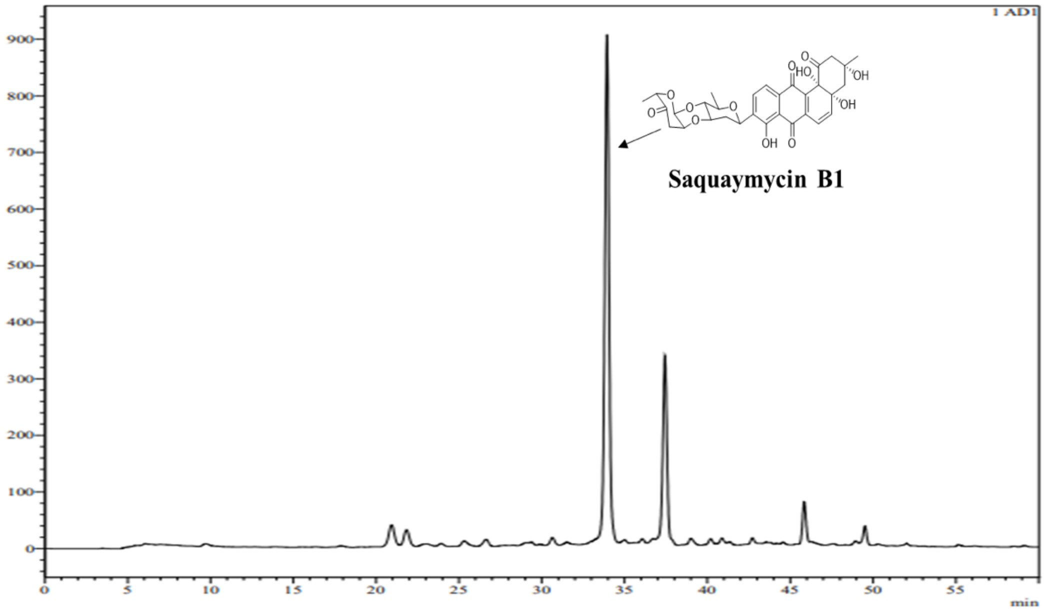

Figure S2 – HRMS spectrum of SQ-B1. The molecular formula and isotopic pattern of the isolated compound are highlighted.

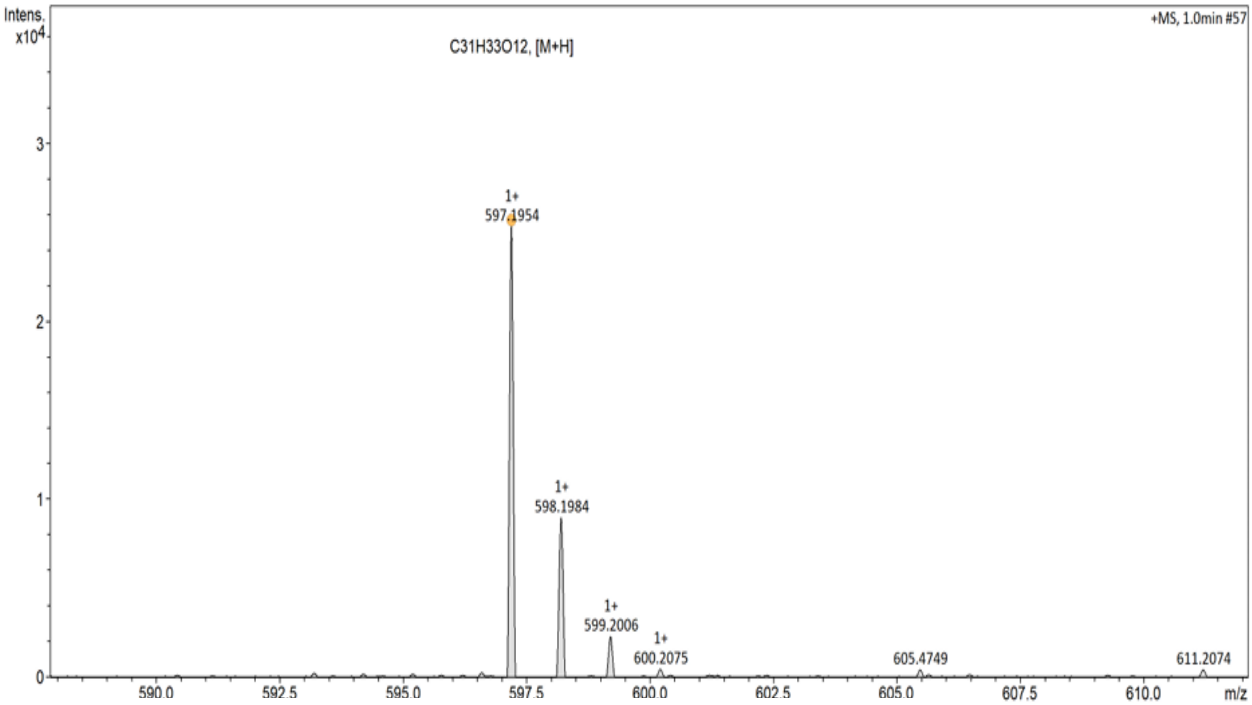

Meas. m/z	#	Ion Formula	Score	m/z	err [mDa]	err [ppm]	mSigma	rdb	e⁻ Conf	N-Rule	Adduct
597.195441	1	C31H33O12	100.00	597.196653	1.2	2.0	6.8	15.5	even	ok	M+H

Figure S3 – Purity analysis of saquayamycin B1 by HPLC. **A:** chromatogram showing the presence of a single peak. **B:** peak purity based on area integration algorithms. **C:** the peak profile, a parameter that indicates the absorption pattern of the compound at different wavelengths, demonstrating that there was no coelution with other detectable compounds. **D:** absorption spectrum of SQ-B1 in the ultraviolet range (190-500 nm).

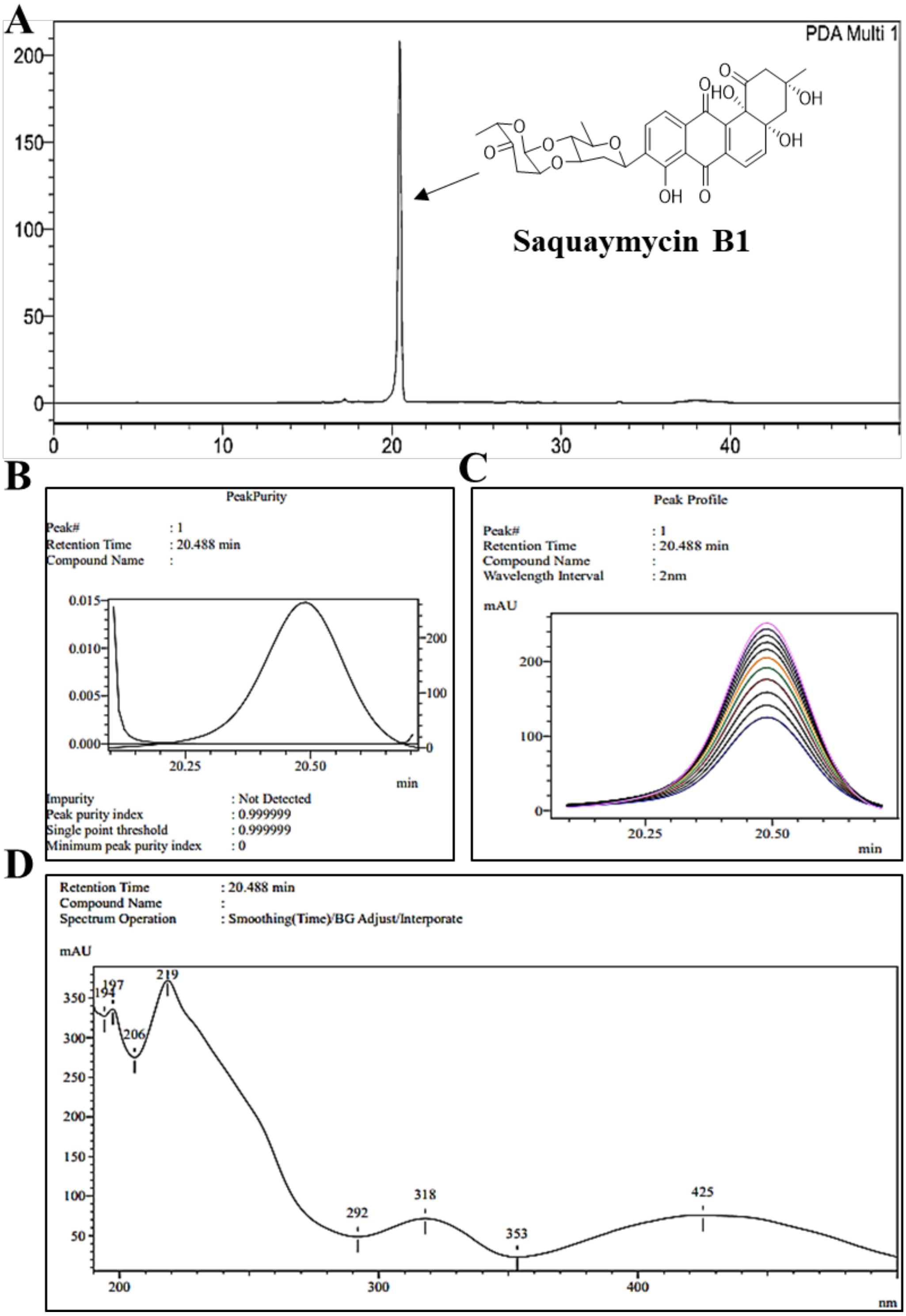

Figure S4 – ^{1}H NMR spectrum, 400 MHZ, in $CDCl_3$.

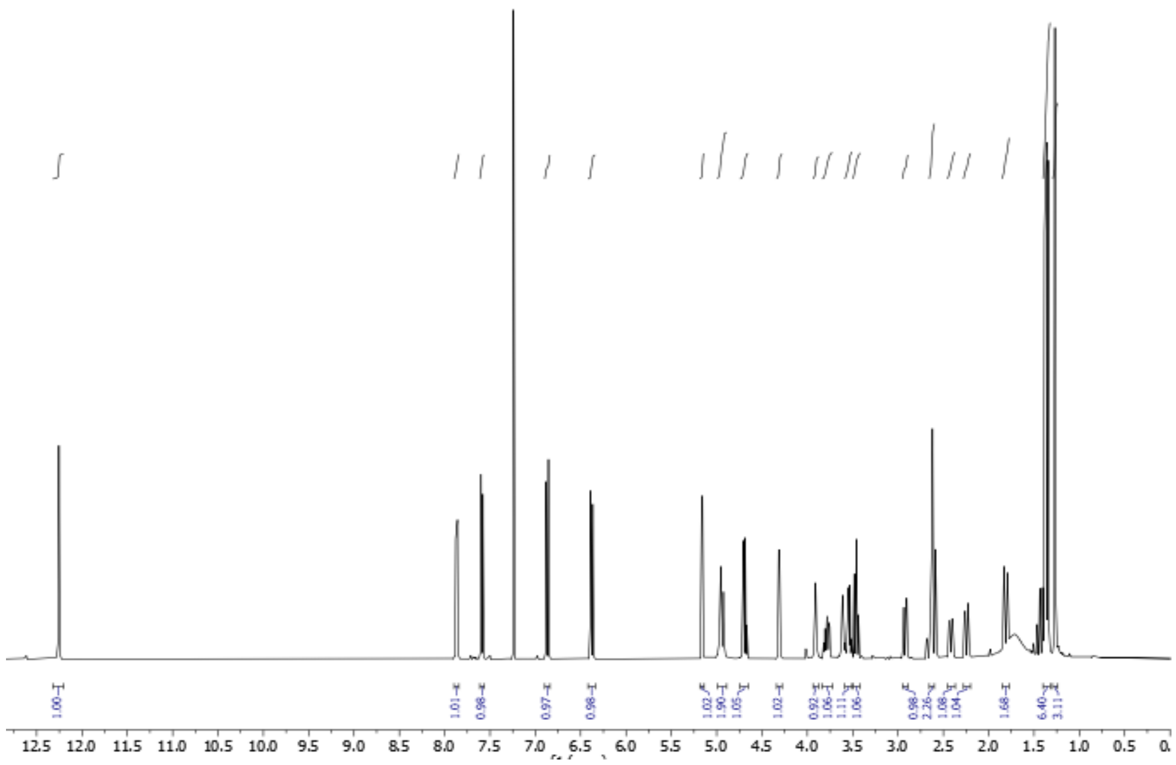

Figure S5 – ^{13}C NMR spectrum, 100 MHZ, in $CDCl_3$.

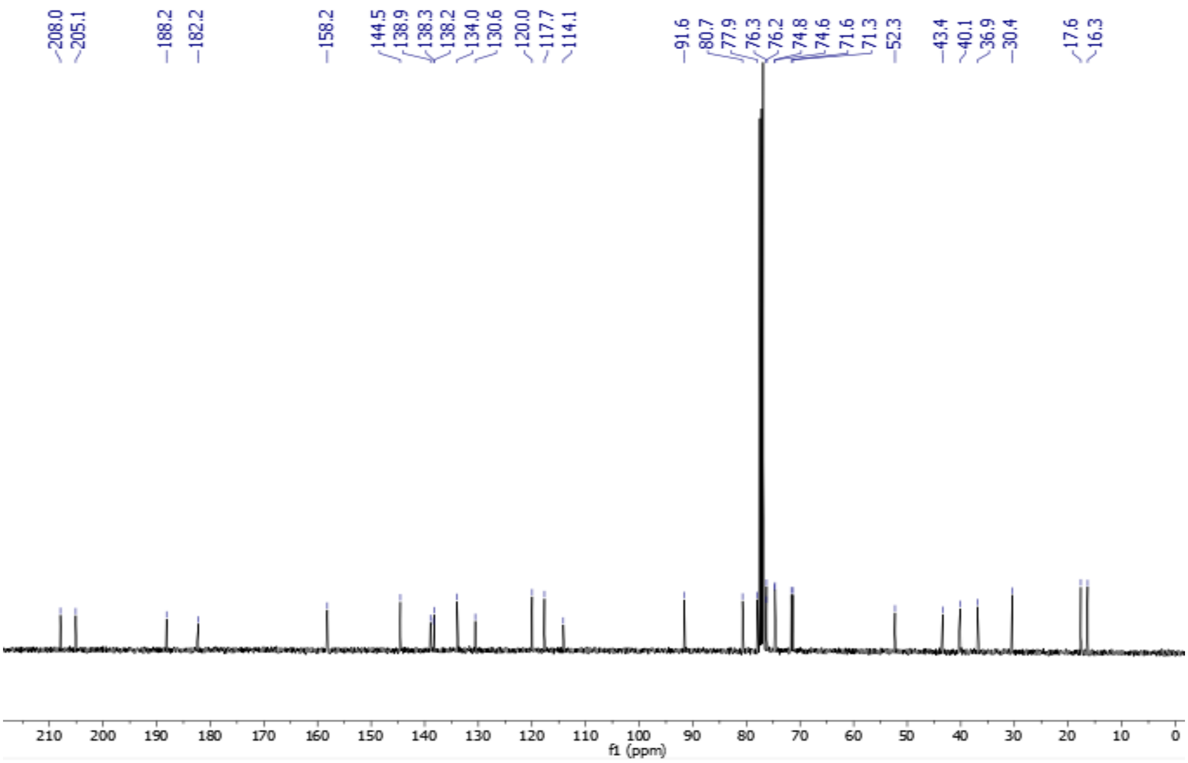

Figure S6 – DEPT spectrum, 100 MHZ, in $CDCl_3$.

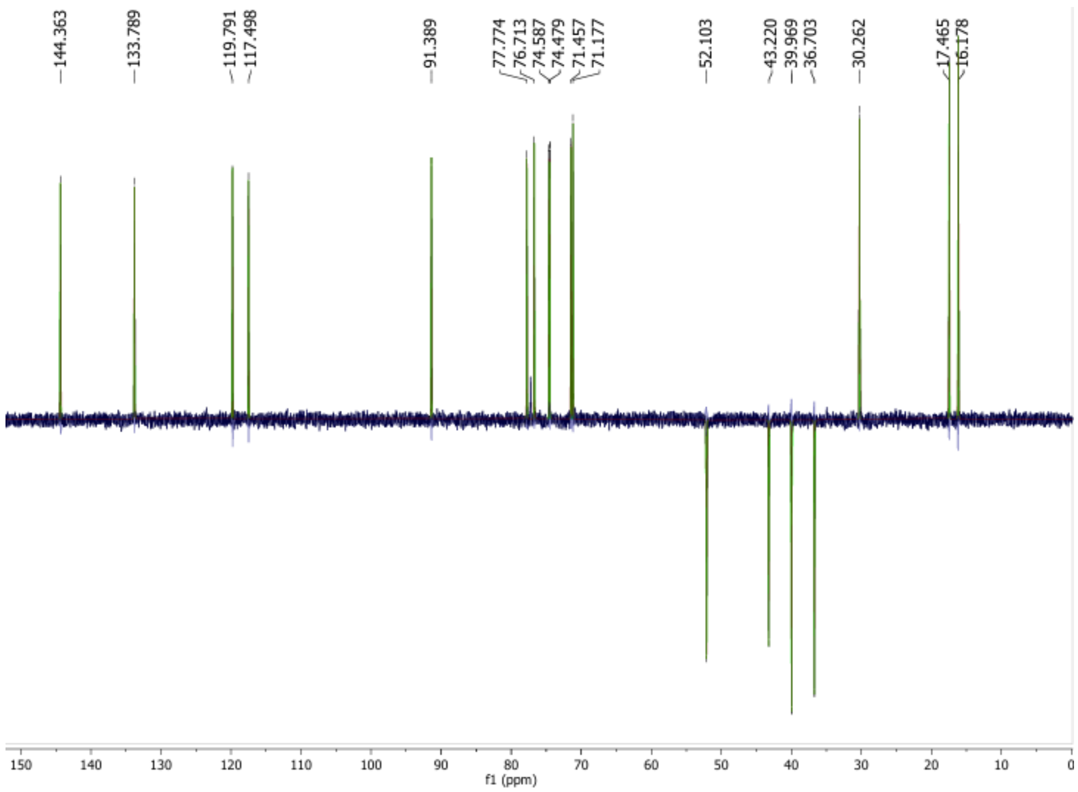

Figure S7 – COSY correlation map, 400 MHZ, in $CDCl_3$.

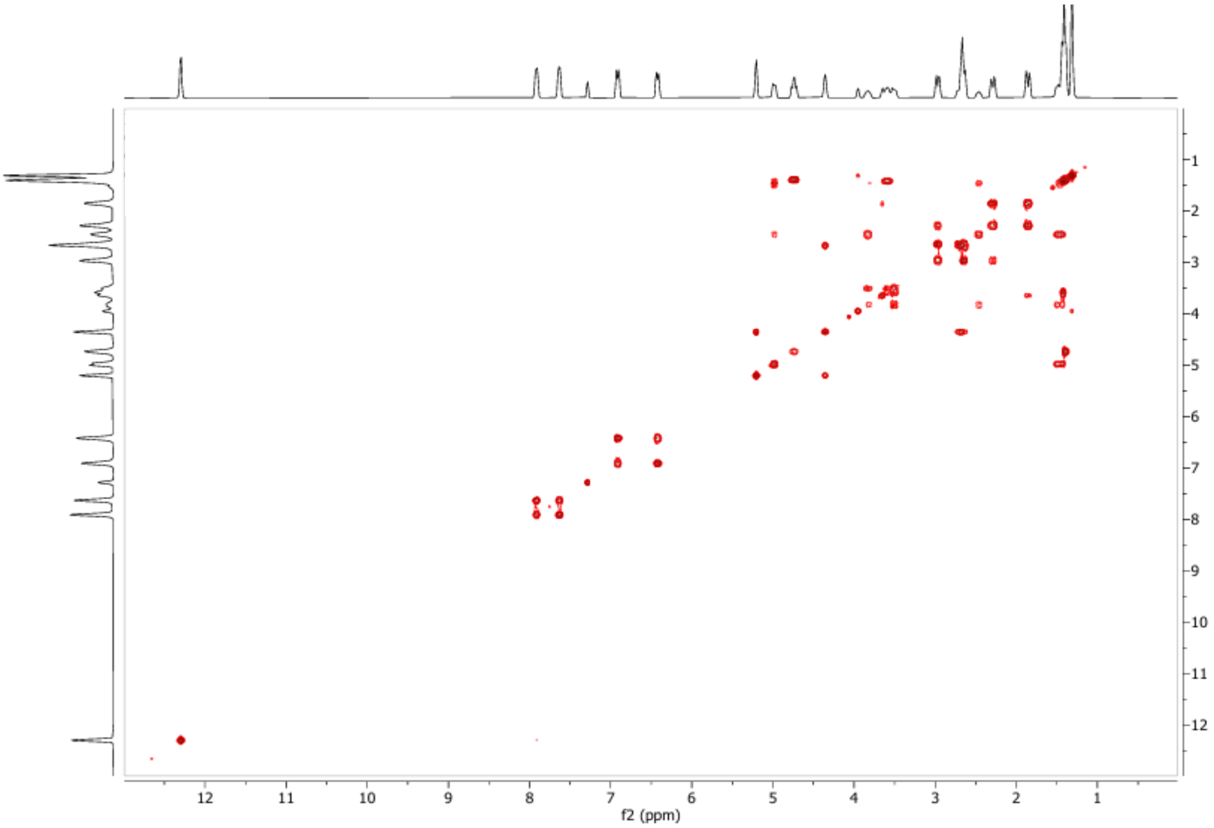

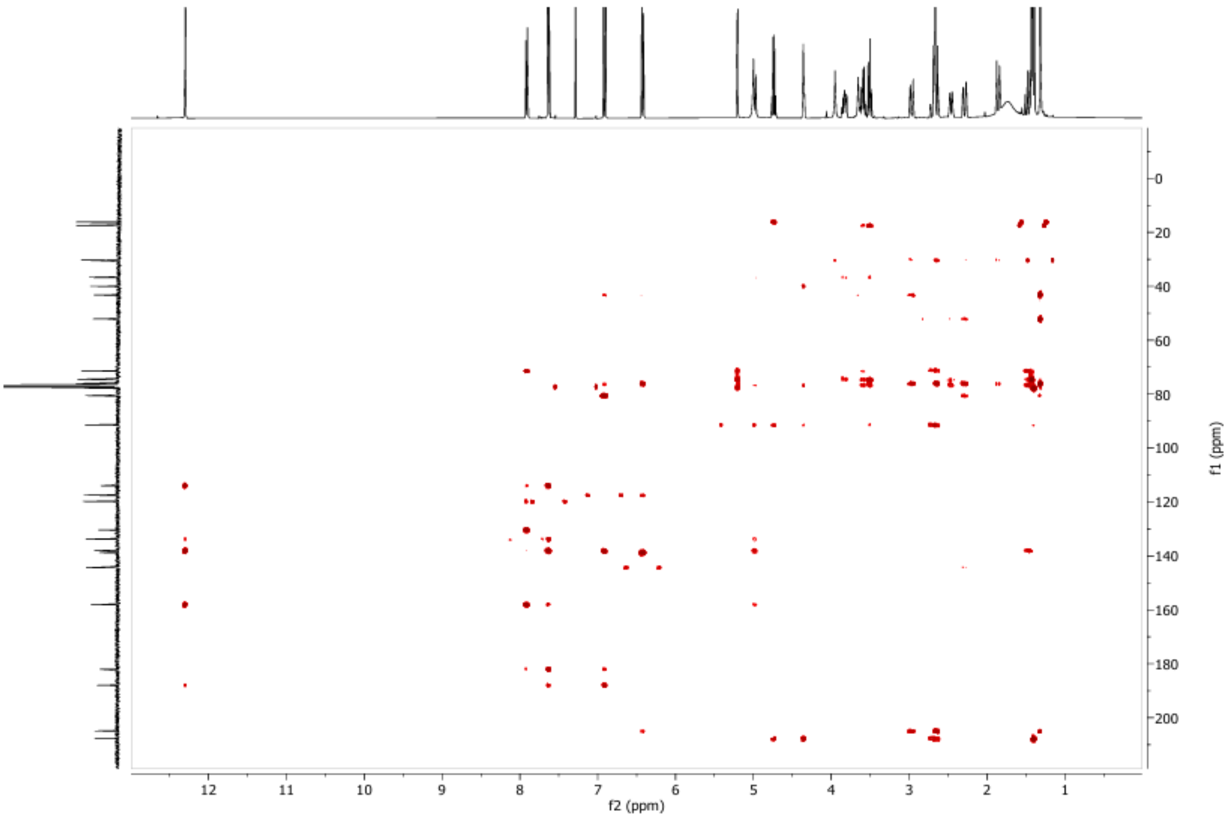

Figure S9 – HSQC correlation map, 400 X 100 MHZ, in $CDCl_3$.

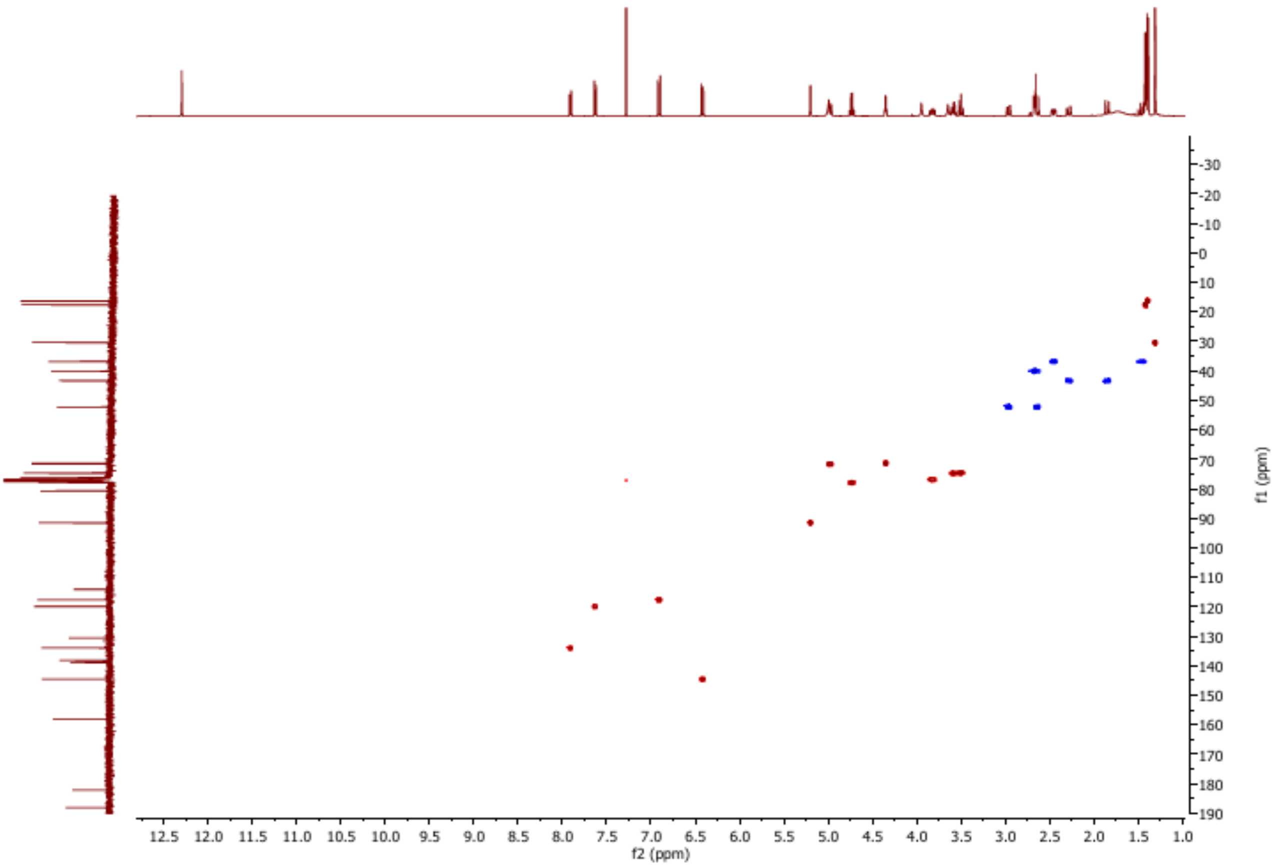

Figure S10 – Fitted dose–response curves for IC_{50} determination of SQ-B1 against human melanoma cell lines and HaCat non-tumor cell line after 24, 48 and 72 h of treatment, by MTT assay.

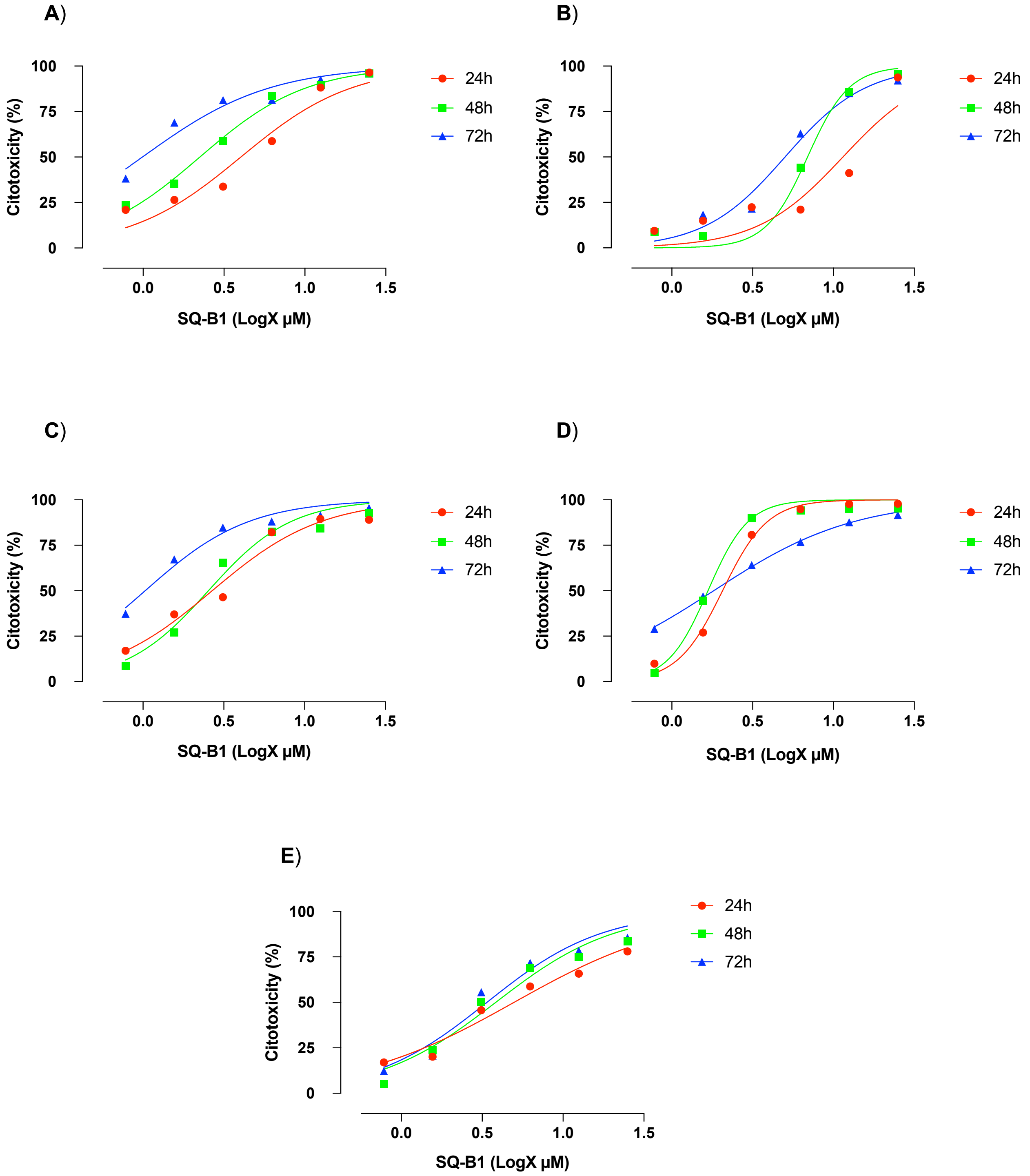

Cells lines were treated with increasing concentrations of SQ-B1 (0.78-25 µM), and cell viability (%) was assessed after 24 h, 48 h, and 72 h of exposure. Data were plotted as a function of $\log_{10}$ concentration (µM) and fitted using a nonlinear regression model with a sigmoidal dose–response (four-parameter logistic) equation. IC_{50} values were calculated from the fitted curves. Data points represent experimental values, and curves correspond to the fitted models. A) SK-MEL-5; B) SK-MEL-113; C) SK-MEL-117; D) SK-MEL-134; E) HaCat.

Figure S11 – Molecular docking analysis of the compound with target proteins: **A/B**. 3D structures GX-pep3 (A) and SQ-B1 (B) against glutathione peroxidase. **C/D**. Amino acid interactions between the same molecules.

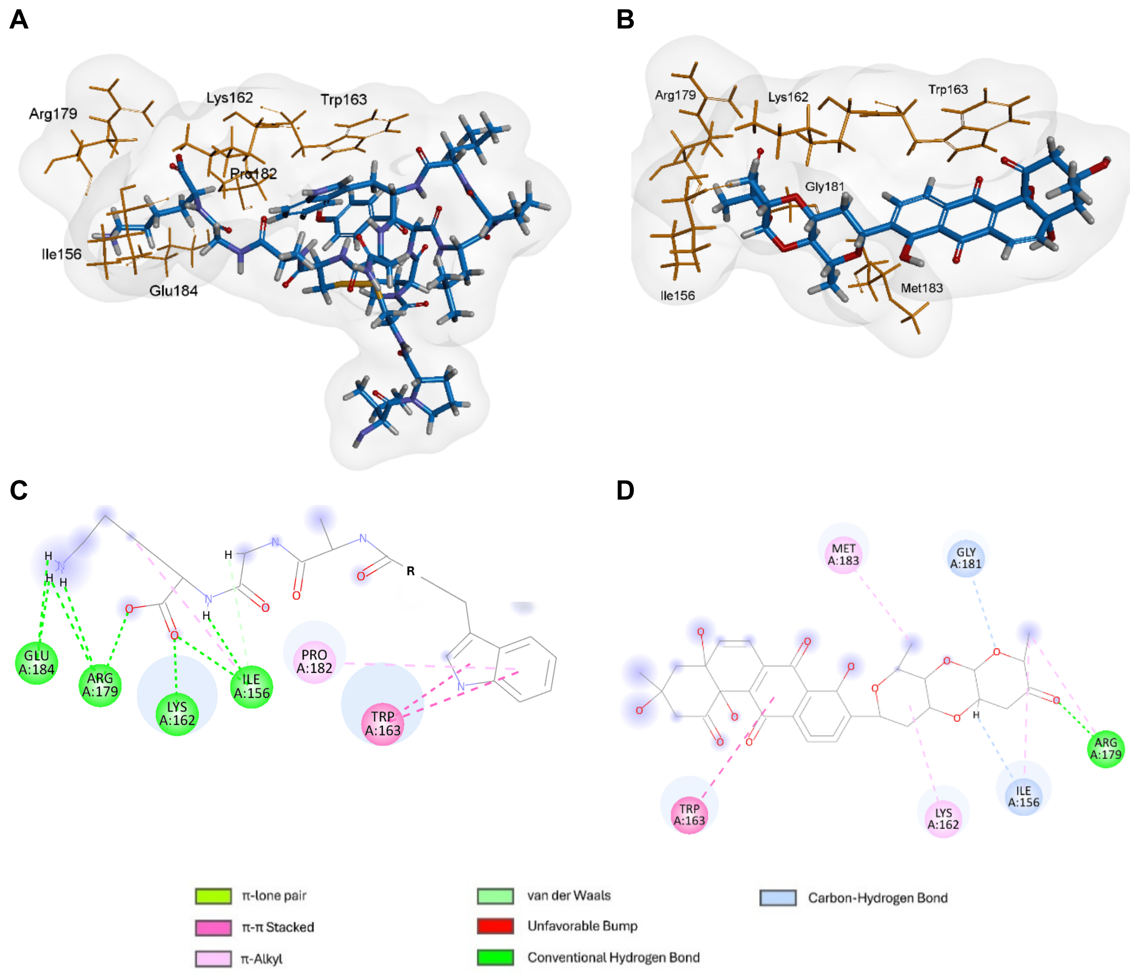

Figure S12 – Molecular docking analysis of the compound with target proteins: **A/B.** 3D structures 6-(7-nitro-2,1,3-benzoxadiazol-4-ylthio)hexanol (A) and SQ-B1 (B) against glutathione transferase. **C/D.** Amino acid interactions between the same molecules.

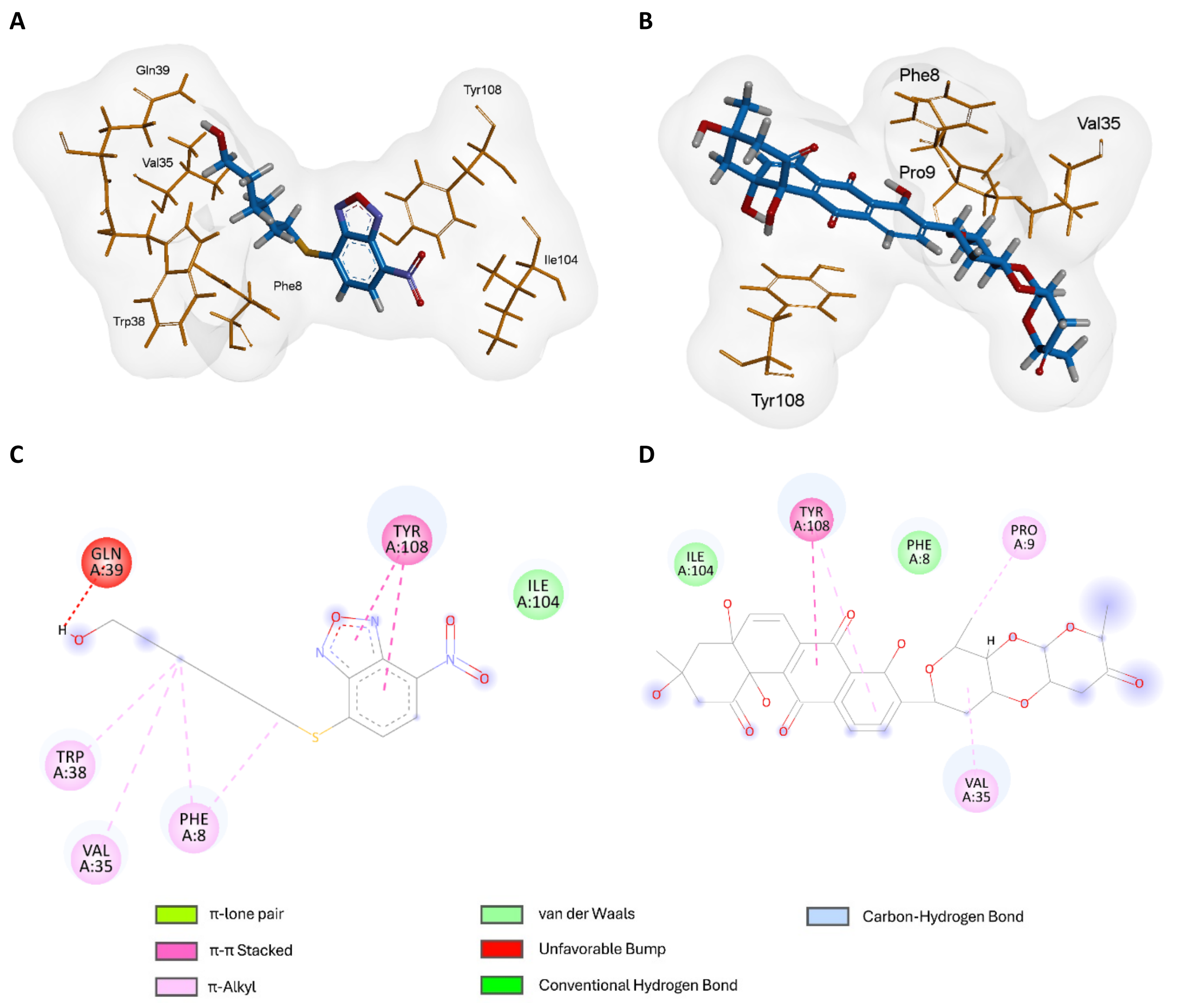

Supplement: Supplementary file 1 — Supplementary Material [file CMDC-21-e70308-s001.pdf]
